# Case Report: Staged treatment of ankle deformity and first metatarsal deficiency after motorcycle spoke injury with Taylor external frame combined with fibular head grafting

**DOI:** 10.3389/fsurg.2024.1391384

**Published:** 2024-09-13

**Authors:** Le Zhang, Yuxin Yan, Pengyu Ren, Yong Hou, Dan Liu, Caiwen Fan, Kehuan Cheng, Jun Zhang, Liangliang Shi

**Affiliations:** ^1^ Department of Surgery II, Jiangxi Phoenix First Hospital, Nanchang, China; ^2^ Department of Orthopedics I, Taiyuan Central Hospital of Shanxi Medical University, Taiyuan, China; ^3^ Department of Neurosurgery, Taiyuan Central Hospital of Shanxi Medical University, Taiyuan, China; ^4^ Department of Plastic Surgery, Nanchang Central Hospital, Nanchang, China; ^5^ Department of Dermatology, The First Affiliated Hospital of Nanchang University, Nanchang, China

**Keywords:** spoke injury, TSF six-axis spatial fixation, free fibular head graft with epiphysis, multi-planar deformities, medial column defects

## Abstract

The treatment of the sequelae of severe foot injuries caused by motorcycle spoke injury, especially in pediatric patients, allows for new options and surgical protocols. The tarsometatarsal joint and the first metatarsal were reconstructed by precise preoperative design using the TSF space external fixation technique in one stage to correct the foot deformity and restore the volume and length, and free grafting of the fibular head with epiphysis in the second stage. This method is the first of its kind reported. The patient’s foot deformity was corrected, walking, walking up and down stairs, and running functions were achieved, and the bone quality could grow with age. The combination of TSF si*x*-axis spatial external fixation technique and microscopic technique can maximize the patient’s appearance and function and is worth promoting.

## Introduction

Motorcycle spoke injury often occurs in countries where bicycles and motorcycles are the main means of transportation. Various reports show that motorcycle spoke injury occurs more frequently in boys between the ages of 2 and 6, and most of the injured parts were found in the heel (1–6). Injuries to the back of the foot and front of the ankle are rarely reported, which may be related to the passengers’ posture. In addition, motorcycles cause more damage because of their higher energy than bicycles.

According to the Oestern and Tscherne classification,soft-tissue injuries can be classified into grade 0 (little or no injury to soft tissue), grade 1(Minor abrasion), grade 2 (Local damage to skin or muscle), and grade 3 (Extensive damage to soft tissue and underlying) (7). Grade 0–1 injuries can be quickly recovered after simple treatment, In patients with grade 2–3 injuries, some severe wounds require flap transplantation to repair the wound surface and it usually takes 1–3 months or more to recover (3). Multiple surgical procedures are required to restore function and weight bearing if the injury results in skin, soft tissue, and bone loss.

We can find some reports of treatments for the injuries combined with skin, soft tissue, Calcaneus, and Achilles tendon, and it can be seen that the treatment is very challenging for the limb salvage (5, 6). However, cases of ankle deformity and loss of the first metatarsal bone after motorcycle spoke injury are rarely reported. That’s what our case is about. The child who suffered motorcycle spoke injury was unable to walk due to an ankle deformity and the absence of the first metatarsal bone after the flap transplant. We report the course and prognosis of the treatment of this patient.

## Case presentation

**Figure 1:**
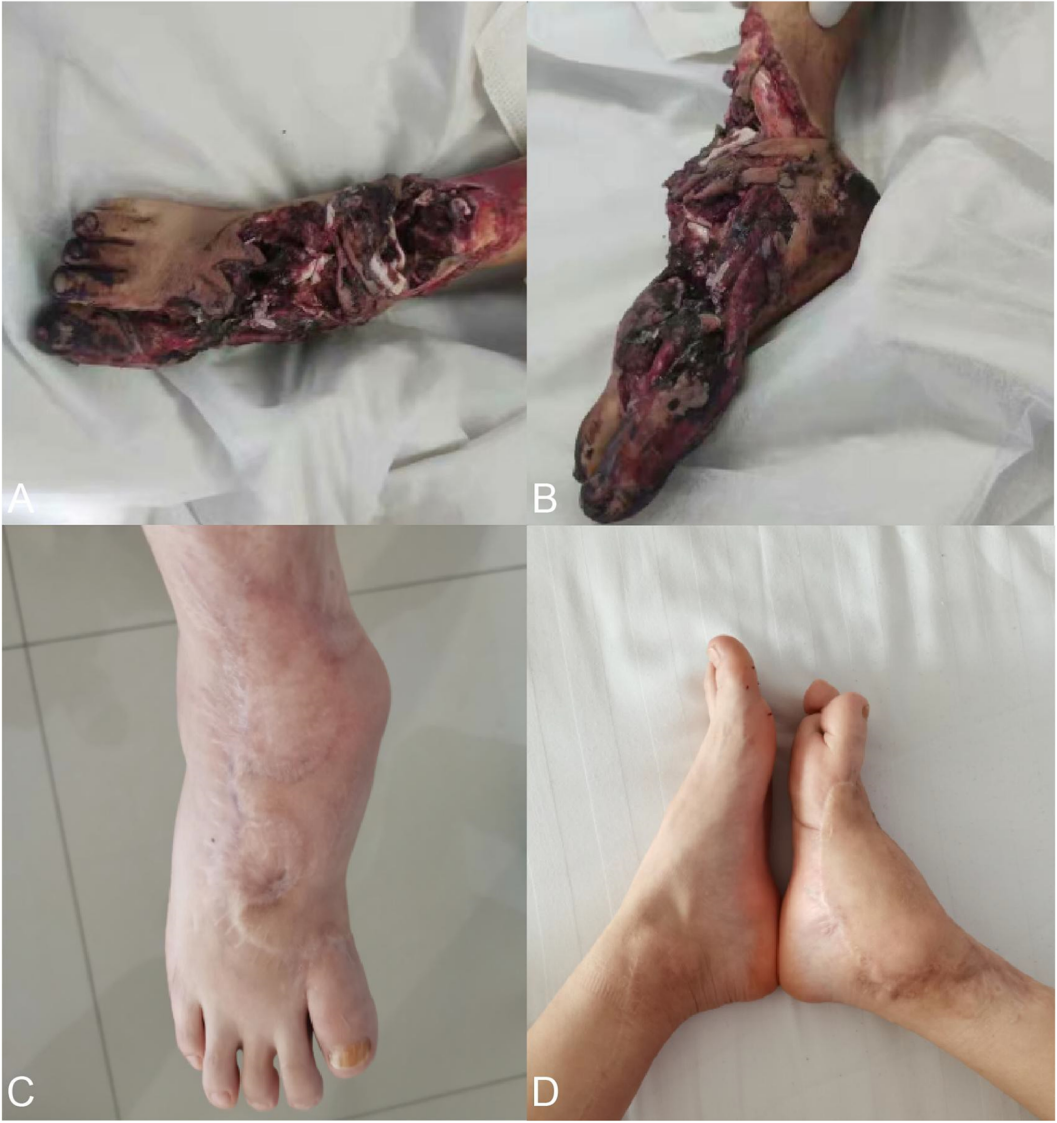
**(A,B)** appearance at other hospitals after an injury. **(C,D)** One year after trauma healing the right foot showed deformity development, acquired flatfoot, and ankle fusion.

An 11-year-old male patient underwent a flap grafting operation one year ago to preserve the limb due to a right foot motorcycle spoke injury. After one year of recovery, the flap survived well, but due to the severe ankle joint injury and the absence of the medial longitudinal arch of the right foot, the child’s right foot gradually developed into a deformity and he walked with a severe limp (Figure 1).

Physical examination: both knees were of the same height, both ankles were unequal in height, internal and external ankle deformities, and no mobility of the ankle joint. The overall length of the foot was about 3 cm shorter than the healthy side, and the first toe of the right foot was floating and unstable due to the absence of the first metatarsal. There was decreased skin sensation on the dorsum of the foot and the dorsal side of each toe, and normal skin sensation on the soles of the feet and the bellies of each toe.

**Figure 2:**
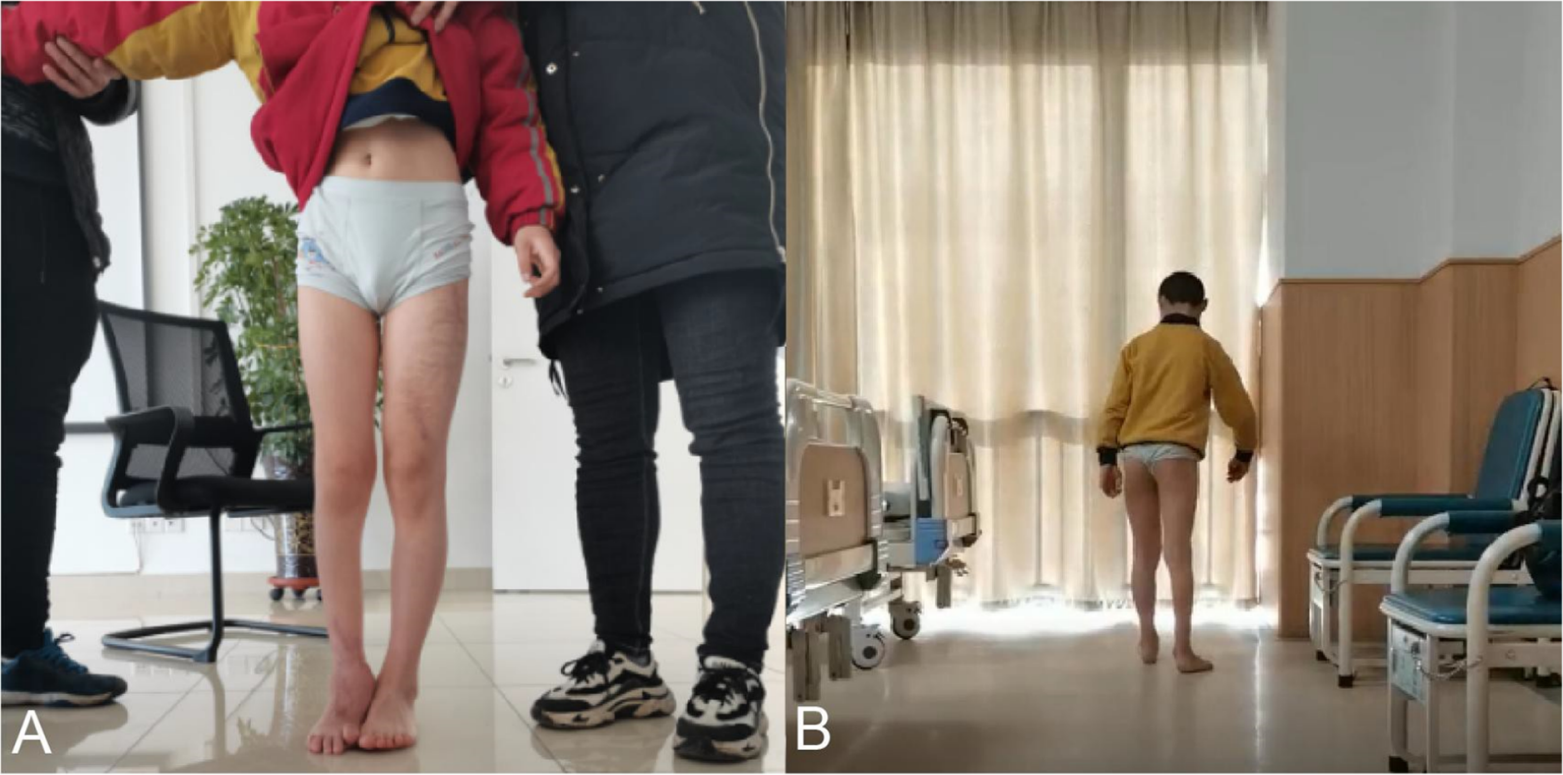
**(A)** The patient needed assistance to stand normally and was unable to touch the floor with his right heel, which needed to be elevated by 2 cm. **(B)** The patient's gait was lame and the right foot was in an externally rotated position.

The child needs to be assisted or have a 2 cm thick plate on the bottom of the right foot to stand up flat on the ground (Figure 2A). The laboratory investigations were unremarkable.

The child had a limp gait with the right foot in an externally rotated position (Figure 2B).

Plain radiographs, CT, and 3D printed models showed that the first metatarsal of the right foot was missing, the proximal part of the second and third metatarsals were missing, a bone bridge was formed with the fourth metatarsal, the navicular bone of the foot was turned toward the sole, and the talus was in an externally turned and internally rotated position, and there was severe osteophytes on all articular surfaces of the ankle joint, with the formation of bone bridges.

### Course of treatment

**Figure 3:**
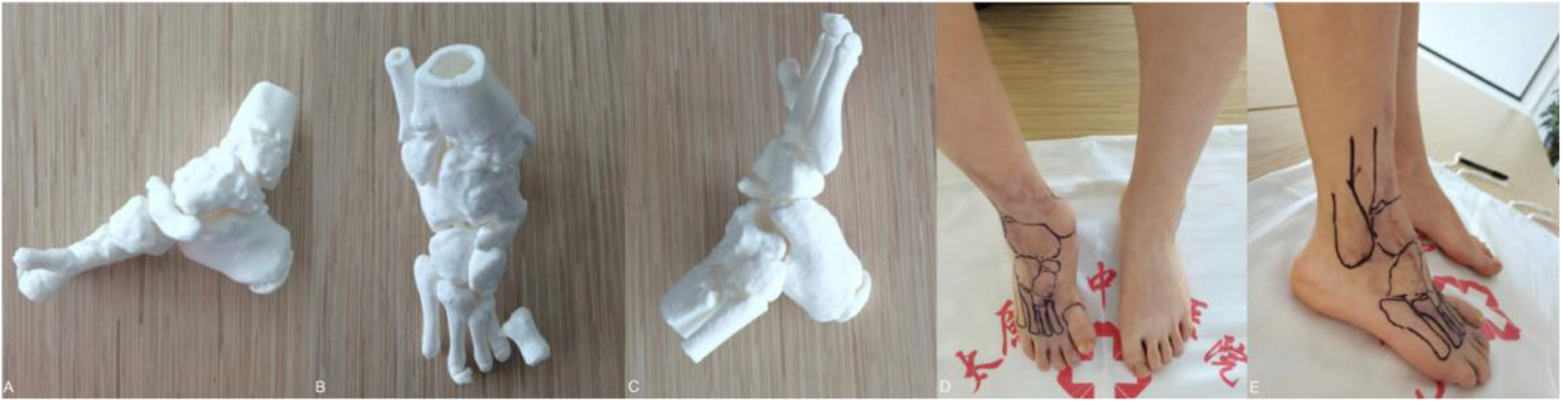
**(A–C)** preoperative 3D printing demonstrates the appearance of bone alignment in the affected foot. **(D,E)** Preoperative marking of the body surface was done according to the 3D print.

We first use a 3D printing model to simulate the affected foot, through the printed 3D model, we can more intuitively observe the distribution of each bone of the affected foot, understand the positional relationship of each bone block, measure the length and size of the missing bone block, and be able to compare it with the healthy side, to better restore the anatomical structure of the affected foot (Figure 3).

The initial surgery was performed using the Taylor six-axis external fixation brace technique with lengthening to correct ankle stiffness as well as valgus deformity, and the ankle joint was debrided to remove the bony bridges and excess bony residue.

**Figure 4:**
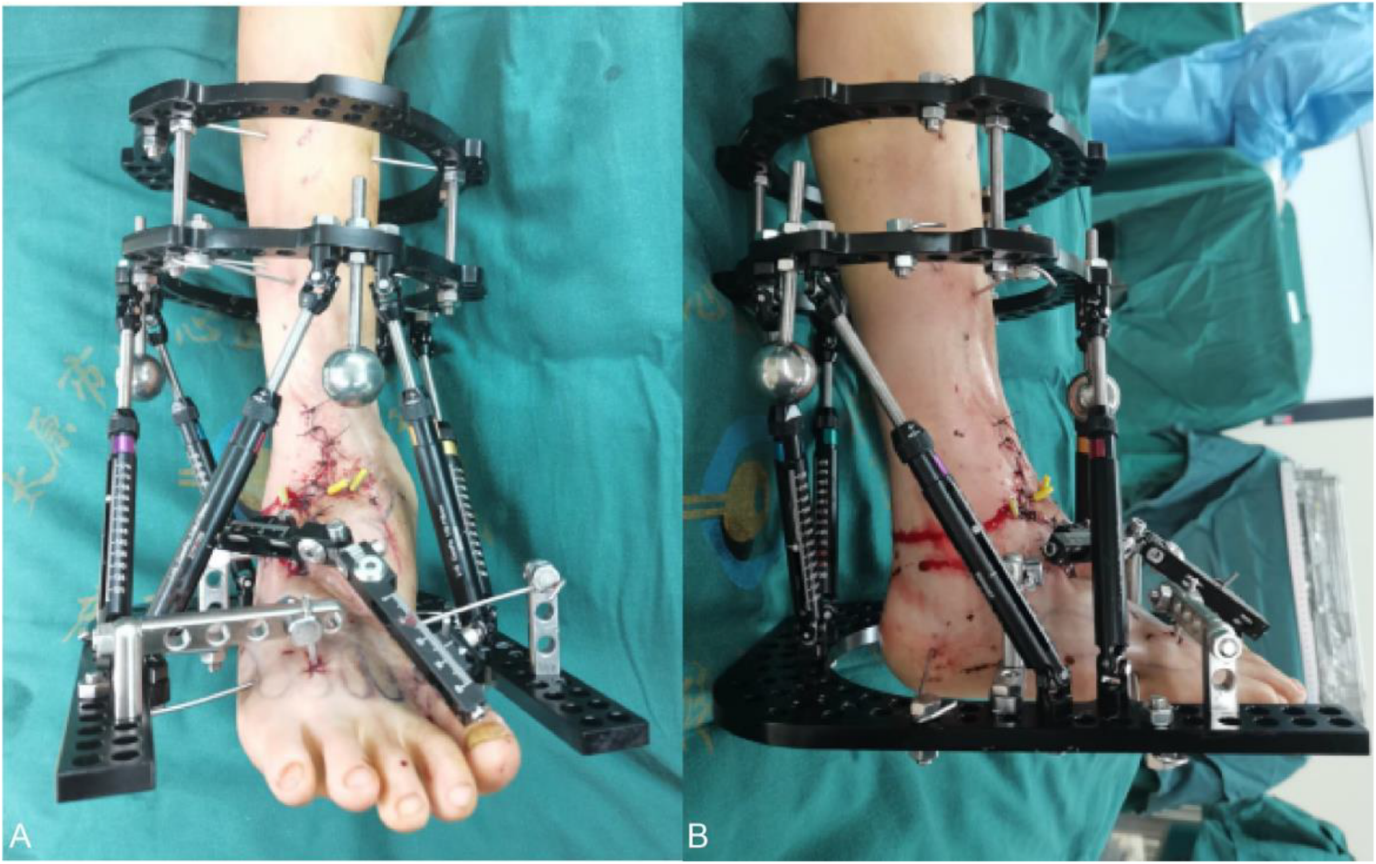
**(A,B)** postoperative appearance after ankle release and si*x*-axis external fixation of TSF, medial volume lengthening.

The main surgical procedure was: two full rings on the lower tibia, the proximal ring was fixed with one 2.0 mm Kirschner wire, the distal ring was fixed with two 2.0 mm Kirschner wires, the heel was fixed with one 2.0 mm Kirschner wire, the talus was fixed with one 4.0 mm Kirschner wire, the distal forefoot was fixed with one 2.0 mm Kirschner wire on the 4 and 5 metatarsals, and the 4th metatarsal was fixed with one 3.0 mm Kirschner wire, and a U-ring was used to fix all the wires. The Taylor bracket was attached and assembled with connecting rods. The first metatarsal was then fixed longitudinally with two 3.0 mm Kirschner wires, and the other 3.0 mm Kirschner wires were fixed to the dice bone with a unilateral extension brace (Figure 4).

**Figure 5:**
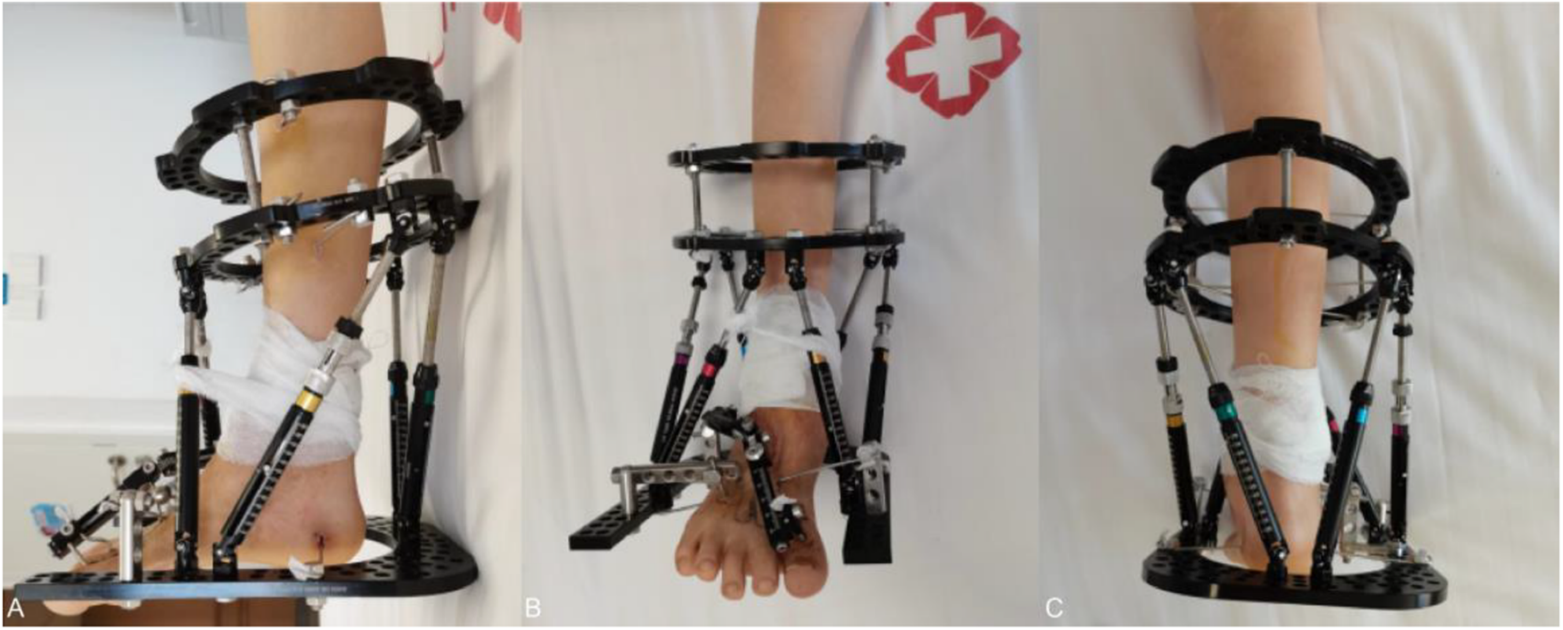
**(A–C)** orthopedic appearance at two months postoperatively, with the ankle close to 90° of flexion and adequate volume in the medial column of the foot.

At the end of the procedure, the child was subjected to a 2-month correction process. The use of the brace lengthening technique lengthens the missing space in the medial aspect of the affected foot and provides for a second repair of the medial column of the affected foot. After the first surgery, the child’s drop foot and valgus deformity were corrected after two months of constant adjustments, and the missing space of the medial column of the foot was adjusted (Figure 5).

**Figure 6:**
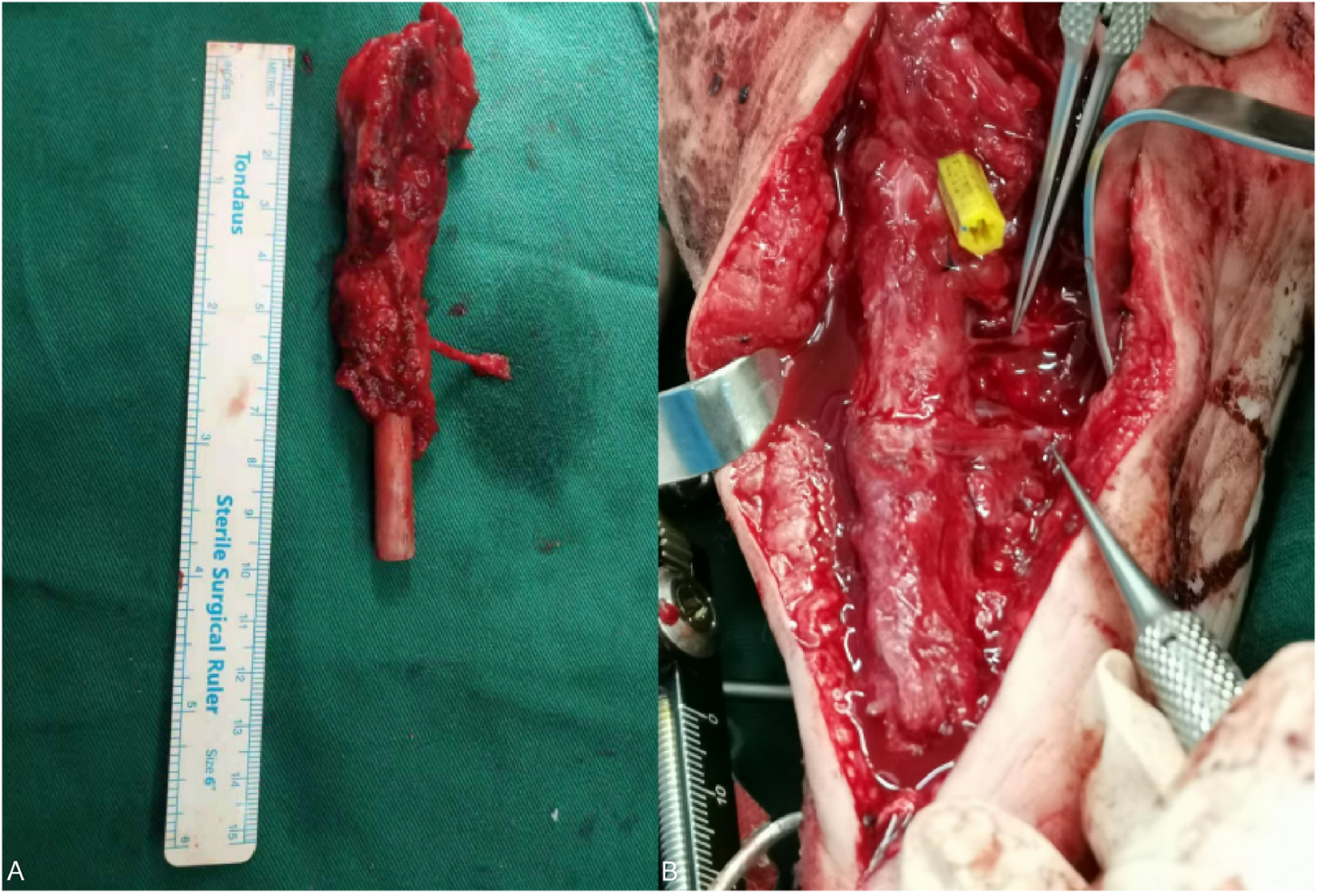
**(A)** Removed epiphyseal fibula head and articular tissue. **(B)** The two supplying vessels of the fibular head are anastomosed to the distal and proximal ends of the medial plantar artery under the microscope.

The TSF was removed at the time of the second surgery and was converted to external fixation with a plaster cast postoperatively as the angle of correction had been satisfactory. A second operation was performed to repair the medial column of the foot and reconstruct the metatarsophalangeal navicular joint using a free graft of the epiphyseal fibular head flap (Figure 6): The length of the fibular head was cut off according to the required length of the fibular head, and the length of the fibular head was about 10 cm, then the medial plantar artery and its accompanying vein were identified, and then the distal and proximal ends of the medial plantar artery and accompanying vein were anastomosed with the two perforating vessels of the fibular head. The fibular head was then fixed with microplates and screws.

**Figure 7:**
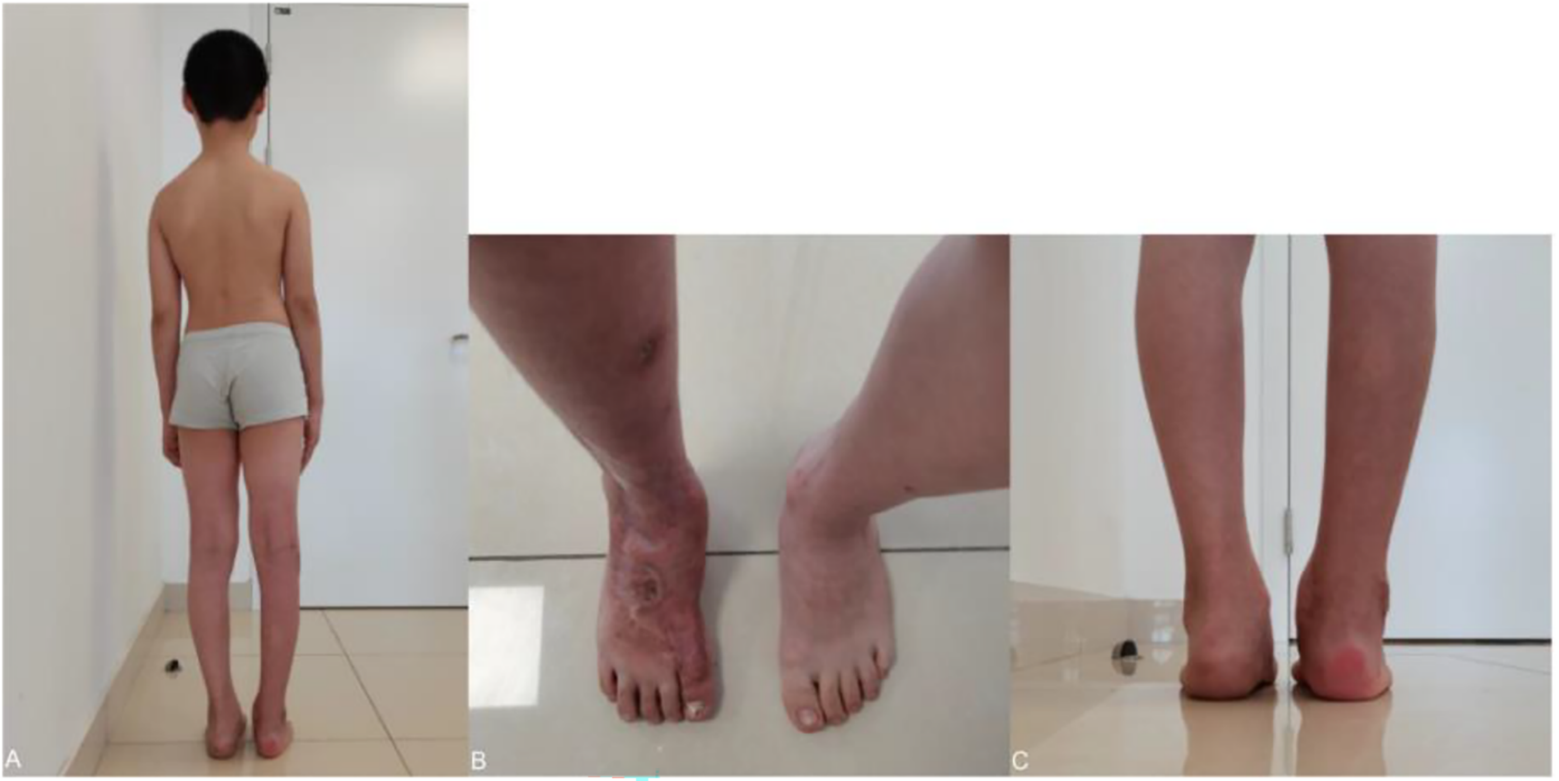
**(A–C)** on review two months after surgery, the patient was able to stand on her own, with both shoulders and both iliacs isometric, and the appearance of the foot was corrected.

This child was able to perform limb rehabilitation exercises in the first month of surgery with the affected limb non-weight-bearing. Two months after the second surgery, we found that the child's standing posture was corrected, the length of the affected foot was equal to that of the healthy side, and the child could walk on his own, with occasional discomfort and no obvious pain (Figure 7). In fact, the child was able to jog even with ankle fusion three months after surgery.

**Figure 8:**
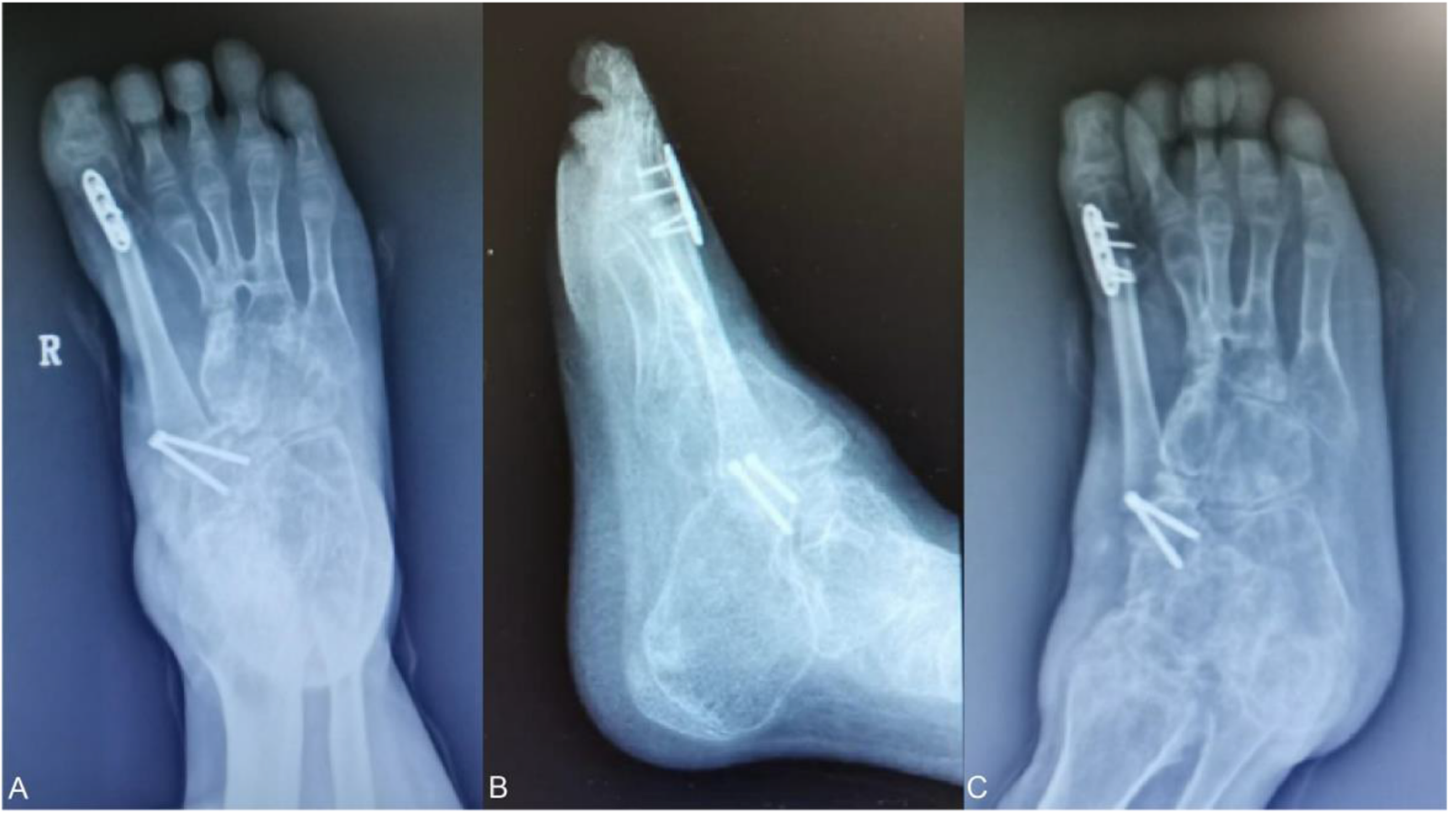
**(A–C)** Two months postoperative x-ray showed that the internal fixation was in place and the medial column reconstruction was in good position.

The x-ray of the foot was reviewed 2 months after the operation, and it was found that the bones of the foot were well repositioned, and the bone quality could grow normally (Figure 8) because the epiphyseal fibular head can continue to grow and develop, so the grafted bone flap is also developing along with the growth of the child.

Posterior radiographs of the heel bone and axial radiographs of the heel bone showed that the exostosis of the affected foot was also appropriately corrected.

Three months after the operation, the patient was re-examined and found that: the patient’s walking had returned to normal this time, and the x-ray showed that the bone had healed well, the joint spaces were in the normal position, the appearance of the affected foot was close to the normal shape, and the deformity was corrected.

Six months postoperative follow-up revealed that the patient was able to perform simple running and jumping maneuvers without significant discomfort.

**Figure 9:**
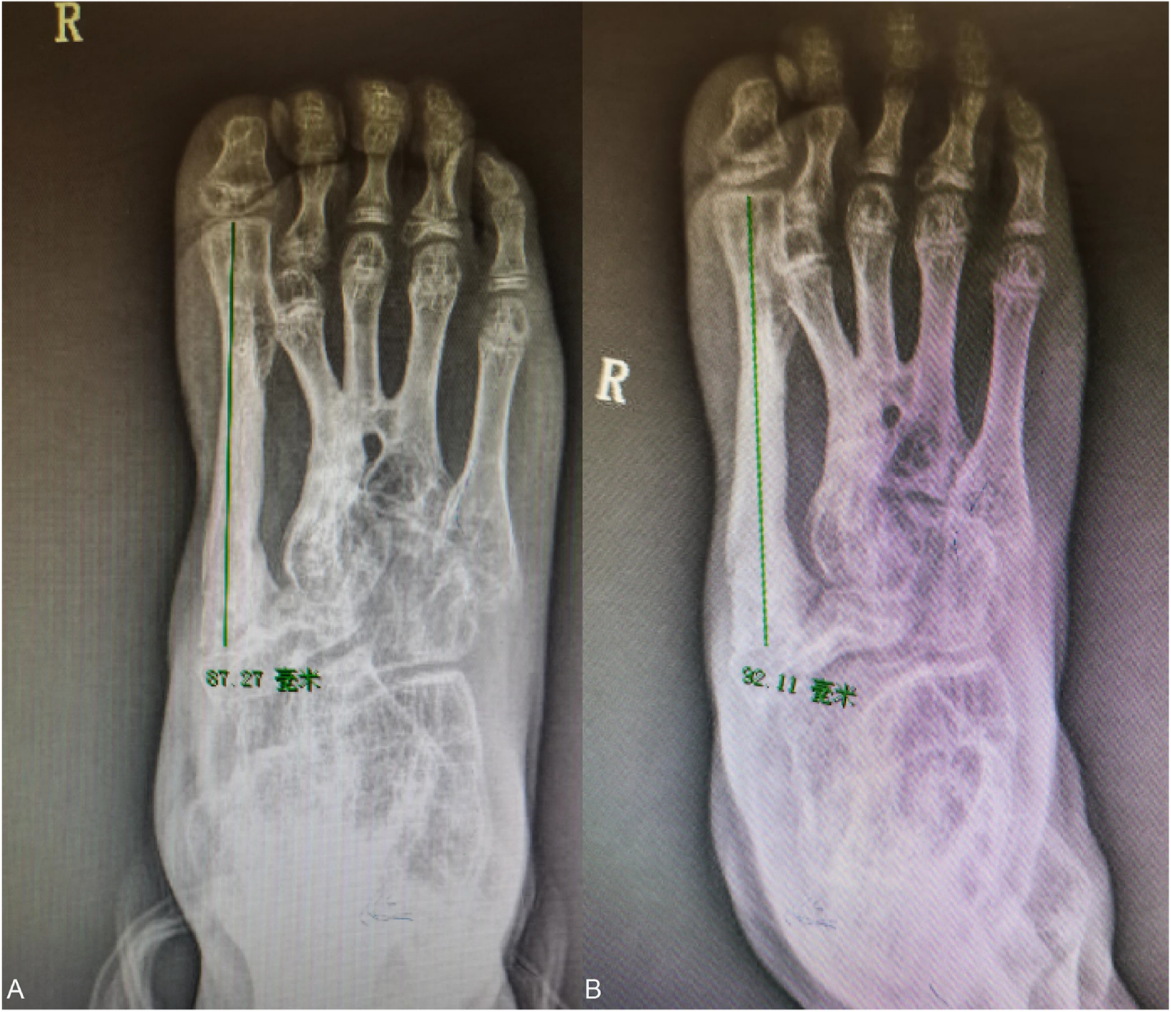
**(A,B)** comparison of grafted bone length in the second six months orthopantomogram (left) and eight months orthopantomogram (right): the first metatarsal train followed the patient's developmental growth by 5 mm.

At the eight-month postoperative follow-up, the walking and running movements were smoother, and the grafted bone had grown about 5 mm compared with the previous one (Figure 9).

## Discussion

Motorcycle spoke injury is a very complex injury, which is mainly caused by the contusion of high-speed rotating wheel spokes, common in children, often accompanied by severe skin, tendon, and bone exposure or even defects, in the face of this complex and serious injury, usually need to be repaired by skin flap grafting, tendon grafting, and in some cases of bone defects need to be carried out by bone removal or even bone flap grafting (5, 6). We know that such injuries often occur in the foot of children (1–3), which is a human structure with complex biomechanical mechanisms that interact with other motor systems. Damage to any structure of the foot can cause changes in the biomechanical mechanisms of other structures. Intact arches are essential for maintaining normal foot function, and children without arches will have a range of serious adverse outcomes (8–10), some patients even lose the opportunity for surgical correction, thus making amputation much more likely.

This child was treated in another hospital and was saved from amputation by covering the wound with a flap, however, the defective foot structure and the damage to the ankle joint still caused an abnormal walking gait in the child. However, this abnormality is progressive, and early restoration of the physiology and mechanics of the foot is the most beneficial treatment for the child.

For multi-planar deformities of the ankle and foot, we chose the Taylor External Fixation Brace technique, guided by cloud computing, to accurately and efficiently correct, drop foot and pronation of the ankle, valgus of the heel bone, and internal rotation of the talus. The shortening of the medial column of the foot brought about by the lack of medial bone in the foot was achieved by using limb distraction techniques to achieve the length we needed. With the development of digital external fixation techniques, the digital si*x*-axis external fixation technique, represented by the TSF, has been increasingly used in clinical limb deformity correction in recent years (11). The frame structure and operation mode are derived from a six-legged parallel robot with degrees of freedom, which has a single structure, better precision and strength than the Ilizarov frame, and a shorter learning curve. Its emphasis on digital analysis of deformity states, assisted by computer simulation and guidance, can enable complex deformities to be synchronized to obtain four-dimensional (3D + spatial) accurate correction, and the TSF shows stronger than the Ilizarov frame under bending and torsion loads. The TSF exhibits greater rigidity than the Ilizarov frame under bending and torsion loads (12–14).

The second problem to be solved is to restore the function of the deformed foot and reconstruct the integrity of the arch. The child’s foot skin has healed, and although there is a large amount of scar tissue formation, the main neurovascular tendons are still present, and there are conditions for bone flap grafting. The choice of graft is a major point of discussion.

Bone grafting has been used in reconstructive surgery for a century, the first free fibula was obtained and transplanted in 1975 by Taylor et al. through a posterior lateral approach, the technique has been popularized by various scholars and recent advances in microvascular technology has made free fibula flap grafting a viable option for the reconstruction of long bone defects (15–17). The shape and size of the fibular head are very similar to that of the missing first metatarsal in this case, and it can fill the missing space, thus maintaining the longitudinal arch stability of the foot and correcting the deformity. In this case, the difference in circumference between the perimeter of the head of the fibula and the perimeter of the proximal first metatarsal joint of the healthy side was less than 1.5 cm. The graft was transplanted to repair the “metatarsal-boat joint”, and it was found that the joint matched well, and the patient did not experience any pain in six months of activity after the operation. In addition, most reports mention the longitudinal growth potential of vascularized epiphyseal grafts (18–21).

After two months, three months, and six months of follow-up, the foot deformity of this case was not only corrected, but also the grafted bone could grow and develop well, which achieved the purpose of this surgery and solved the problem of walking difficulty for the patient, but it is worth noting that the epiphyseal closure of the fibular head is different from that of metatarsal bones, so we should pay close attention to this difference in the follow-up. In addition, the growth rate of the fibular head is different from that of the other metatarsals, so it is important to monitor the changes in the length of the bones in the child’s foot and to intervene in a timely and appropriate manner when necessary to avoid recurrence of deformity. Repair of longitudinal arch deficiency is still a challenge in orthopedics, and the use of fibular head transplantation for reconstruction of the medial column of the foot is rare and needs to be explored; also, to ensure the blood circulation for fibular head growth, anastomosis of blood vessels is required by ultramicrographic techniques, which increases the risk of the surgery.

Although a definitive repair was obtained in this case, many problems still exist, such as secondary deformity under long-term stress, osteoarthritis, and chronic pain are still something we need to explore in the future. In addition, the shortcoming of this case is that the ankle fusion was not detected early in the first stage of limb preservation and rehabilitation in an outside hospital, which resulted in the child’s ankle still starting to fuse again after the first surgery in this admission to the rehabilitation process, and the ankle mobility continued to decrease. The patient’s parents were already very satisfied with the treatment and refused to undergo another surgery because they were worried that another surgery would aggravate the child’s psychic injury, which was a pity.

## Conclusion

Treatment of severe motorcycle spoke injury varies on an individualized basis, depending on the extent of tissue loss after the injury. Since most motorcycle spoke injury are sustained by children, major injuries can result in premature disability and limited mobility. Fortunately, with advances in surgical technology, we can use better tools and more advanced techniques to preserve as much of the limb as possible and restore the patient’s normal mobility. The advantage of our surgery is that we are fully familiar with the anatomy of the limb, and we use the TSF 6-axis external fixation technique and its cloud computing ability to accurately correct the alignment and structure of the bone, and we use microscopic techniques to graft the missing bone and epiphyses, to achieve the “make up for what is lacking” requirement, and at the same time, to maximize the restoration of the patient’s appearance. This combined surgical approach deserves to be promoted in orthopedics and micrografting after severe trauma and congenital defects, especially in children with growing bone, where the grafted bone can grow with age.

## Data Availability

The raw data supporting the conclusions of this article will be made available by the authors, without undue reservation.
